# In Vitro Antioxidant, Antibacterial, and Cholinesterase‐Inhibitory Properties of an Aqueous *Euphorbia macroclada* Root Extract Obtained by Ultrasound‐Assisted Extraction

**DOI:** 10.1002/cbdv.71825

**Published:** 2026-10-12

**Authors:** Abdulkadir Gül, Fırat Yılmaz, Fevzi Topal, İnanç Özgen, Ercan Aydoğmuş, Hasan Arslanoğlu

**Affiliations:** ^1^ Department of Emergency Aid and Disaster Management, Faculty of Health Sciences Gümüşhane University Gümüşhane Türkiye; ^2^ Department of Food Engineering, Faculty of Engineering and Natural Sciences Gümüşhane University Gümüşhane Türkiye; ^3^ Department of Nutrition and Dietetics, Faculty of Health Sciences Gümüşhane University Gümüşhane Türkiye; ^4^ Department of Bioengineering, Faculty of Engineering Fırat University Elazığ Türkiye; ^5^ Department of Chemical Engineering, Faculty of Engineering Fırat University Elazığ Türkiye; ^6^ Department of Materials Science and Engineering University of North Texas Denton Texas USA; ^7^ Department of Chemical Engineering, Faculty of Engineering Çanakkale Onsekiz Mart University Çanakkale Türkiye

**Keywords:** Alzheimer's disease, antibacterial activity, anticholinesterase activity, *Euphorbia macroclada*, ultrasound‐assisted extraction

## Abstract

In the present study, the aqueous extract obtained from the roots of *Euphorbia macroclada* via ultrasound‐assisted extraction (Ult‐ER) was evaluated for its anticholinesterase (AChE and BChE), antioxidant, and antibacterial activities. While the extract exhibited weak inhibition against AChE (IC_50_: 507.69 ± 17.95 mg/mL), it showed relatively greater inhibitory activity against BChE (IC_50_: 22.41 ± 0.49 mg/mL). In antioxidant assays, Ult‐ER exhibited measurable antioxidant responses, with DPPH and ABTS^+^ radical‐scavenging capacities of 18.33 ± 0.49 and 119.59 ± 1.86 mg TE/L, respectively. Its Fe^3^^+^ and Cu^2^^+^ reducing capacities were 128.51 ± 12.57 and 670.33 ± 11.59 mg TE/L, respectively. The total phenolic and flavonoid contents were 84.80 ± 3.31 mg GAE/g and 60.67 ± 2.29 mg QE/g, while the total antioxidant capacity was 9.78 ± 0.44 mg TE/g and 15.62 ± 1.85 mg BHAE/g. In the antibacterial evaluation, Ult‐ER at the highest tested concentration of 8 mg/mL exhibited inhibitory activity against all tested strains, including *Enterococcus faecalis* ATCC 29212, *Staphylococcus aureus* ATCC 25923, *Escherichia coli* ATCC 25922, and *Pseudomonas aeruginosa* ATCC 27853, whereas no detectable inhibition was observed at lower concentrations.

## Graphical abstract

The aqueous root extract of *Euphorbia macroclada* was obtained using ultrasound‐assisted extraction (UAE). The extract showed remarkable butyrylcholinesterase (BChE) inhibition relevant to Alzheimer's disease. Strong antioxidant capacity was demonstrated through multiple radical scavenging and reducing assays. High phenolic and flavonoid contents contributed to the observed bioactivities. The extract showed broad‐spectrum antibacterial activity against Gram‐positive and Gram‐negative bacteria.

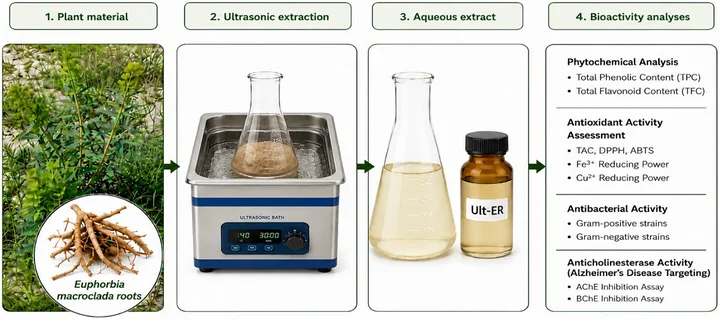

## 1 Introduction

Alzheimer's disease (AD) is the most prevalent cause of dementia worldwide and represents a major public health concern characterized by progressive and irreversible neurodegenerative alterations in the central nervous system [1]. The disease predominantly affects individuals over 65 years of age and manifests clinically with memory loss, cognitive decline, language and orientation impairments, behavioral changes, and difficulties in performing activities of daily living [2]. Globally, dementia affects approximately 46 million people, and this number is projected to reach 131 million by 2050. In the absence of effective preventive or disease‐modifying therapies, this increase is expected to accelerate further ([3]; Alzheimer's Association [4]). Although the exact etiology of AD has not been fully elucidated, the accumulation of *β*‐amyloid plaques, formation of neurofibrillary tangles, increased oxidative stress, mitochondrial dysfunction, and impairments in cholinergic neurotransmission are recognized as key pathological mechanisms underlying the disease [5]. In particular, degeneration of cholinergic neurons in the basal forebrain leads to a marked reduction in acetylcholine levels, thereby accelerating cognitive decline [6]. Acetylcholine is one of the principal neurotransmitters involved in learning and memory processes [7]. Its hydrolysis in the synaptic cleft is catalyzed by acetylcholinesterase (AChE) and butyrylcholinesterase (BChE), and excessive activity of these enzymes further exacerbates the neurodegenerative process [8, 9].

Currently, there is no definitive cure for AD, and existing pharmacological approaches provide only limited symptomatic relief [10]. Approved AChE inhibitors for clinical use include galantamine, rivastigmine, tacrine, and donepezil, most of which are effective primarily in mild to moderate stages of AD [11]. However, the occurrence of adverse effects such as gastrointestinal disturbances, hepatotoxicity, dizziness, and nausea, along with pharmacokinetic limitations associated with long‐term use, necessitates the continued search for novel and safer therapeutic agents [12].

Oxidative stress results from an imbalance between reactive oxygen species (ROS) production and antioxidant defense systems [13]. In recent years, the central role of oxidative stress in the pathogenesis of AD has gained increasing recognition [14]. An imbalance between excessive ROS generation and cellular antioxidant defense mechanisms leads to lipid peroxidation, protein oxidation, and DNA damage in neuronal cells, thereby accelerating the neurodegenerative process [15, 16]. Therefore, multifunctional therapeutic strategies targeting both cholinesterase inhibition and suppression of oxidative stress are considered promising approaches in the management of AD [1]. Antioxidants are molecules that mitigate oxidative damage by stabilizing free radicals and play a significant role in slowing the progression of chronic diseases [17]. The literature indicates that natural compounds with antioxidant properties may exert synergistic neuroprotective effects when used in combination with AChE inhibitors [18, 19]. In this context, medicinal plants have attracted increasing attention as natural alternatives to synthetic drugs due to their potent antioxidant and cholinesterase inhibitory potential [20].

Nowadays, not only neurodegenerative diseases but also antibiotic resistance pose serious global health threats. Antimicrobial resistance (AMR) arises when pathogenic microorganisms develop resistance to existing treatments as a result of widespread and uncontrolled antibiotic use, making infection control increasingly difficult [21]. According to the World Health Organization (WHO) 2024 Global Antimicrobial Resistance Report, resistance increases were observed in more than 40% of antibiotic–pathogen combinations monitored between 2018 and 2023 [22]. This situation has further emphasized the importance of investigating new biologically active compounds from natural sources [23]. In light of these multifaceted health challenges, medicinal plants have emerged as important natural resources for the development of novel therapeutic agents [24].

The *Euphorbiaceae* family is notable for its extensive diversity, comprising approximately 300 genera and 8000 species, as well as its rich phytochemical profile [25]. Particularly abundant in alkaloids, terpenoids, and phenolic compounds, this family includes numerous species with diverse pharmacological properties [26]. The genus *Euphorbia*, with more than 1800 species, is one of the largest genera of flowering plants and is widely represented in the flora of Türkiye under the common name “sütleğen” [25, 26]. *Euphorbia* species have traditionally been used in folk medicine for the treatment of migraine, skin disorders, parasitic infections, and warts, while modern scientific studies have confirmed their cytotoxic, antitumor, antibacterial, anti‐inflammatory, and antioxidant activities [27, 28, 29]. One of the notable members of this genus, *Euphorbia macroclada* (*E. macroclada*), is widely distributed across Central, Eastern, and Southeastern Anatolia and is recognized for its high adaptability to diverse environmental conditions [30]. Various parts of the plant have been reported to contain polyphenols, flavonoids, tannins, alkaloids, saponins, and terpenoid compounds [31].

Although numerous studies have investigated the therapeutic applications of *Euphorbia* species [30, 32, 33, 34], information remains limited regarding the combined evaluation of cholinesterase‐inhibitory activity relevant to Alzheimer's disease together with the antioxidant and antibacterial activities of the aqueous extract obtained from the roots of *E. macroclada* via ultrasound‐assisted extraction (UAE). Accordingly, the present study aims to contribute to addressing this scientific gap. In this context, the aqueous extract derived from the roots of *E. macroclada* was evaluated for its anticholinesterase, antioxidant, and antibacterial activities, along with its total phenolic content (TPC) and total flavonoid content (TFC). The TPC and TFC of the extract were estimated using the corresponding colorimetric assays. Antioxidant capacity was assessed through total antioxidant capacity (TAC), DPPH (2,2‐diphenyl‐1‐picrylhydrazyl), and ABTS [2,2′‐azino‐bis(3‐ethylbenzothiazoline‐6‐sulfonic acid)] radical scavenging assays, as well as Fe^3+^ and Cu^2+^ reducing power assays. In addition, the antibacterial activity of the extract was evaluated against Gram‐positive (*E. faecalis* and *S. aureus*) and Gram‐negative (*E. coli* and *P. aeruginosa*) bacterial strains. One contribution of this study is the combined evaluation of these biological properties in the same aqueous root extract. In this regard, the anticholinesterase activity of the extract was determined in vitro against AChE and BChE enzymes using spectrophotometric methods. Overall, this study provides an integrated in vitro evaluation of the anticholinesterase, antioxidant, and antibacterial activities of the aqueous extract obtained from *E. macroclada* roots via UAE, providing for further investigation of the extract's bioactive constituents and cholinesterase‐inhibitory properties.

## 2 Materials and Methods

### 2.1 Materials

In this study, the roots of *E. macroclada* were used as plant material. The plant material was collected in July 2024 from Tekke Village, located in the central district of Gümüşhane, Türkiye (40.417690 N, 39.585003 E). The material was collected from its natural habitat and was not commercially purchased. Taxonomic identification of the collected specimens was performed by Prof. Dr. İnanç Özgen, Department of Bioengineering, Fırat University, Türkiye. A representative voucher specimen was prepared and deposited in the Fırat University Department of Pharmacy Herbarium, Elazığ, Türkiye, under the voucher number FUE003. After collection, the plant material was initially washed with tap water and subsequently rinsed several times with deionized water to remove adhering soil, dust, and other impurities. The samples were then air‐dried in the dark at room temperature in a well‐ventilated environment for approximately 10 days to ensure complete drying. At the end of this period, the roots were separated, cut into small pieces, and ground into a fine powder using an electric blender (Blender 8011ES, USA). The powdered root material was stored in airtight amber glass containers at 4°C until extraction. All chemicals used in the antioxidant, antibacterial, and enzyme inhibition assays were purchased from Merck KGaA (Darmstadt, Germany), and Sigma–Aldrich (St. Louis, MO, AND). Deionized water was used throughout all experimental procedures and for the preparation of solutions.

### 2.2 Preparation of the Plant Extract

In this study, the extract obtained from *E. macroclada* was prepared to evaluate its antioxidant, antibacterial, and inhibitory effects on AChE and BChE enzymes, and the experimental workflow is presented in Figure 1.

**FIGURE 1:**
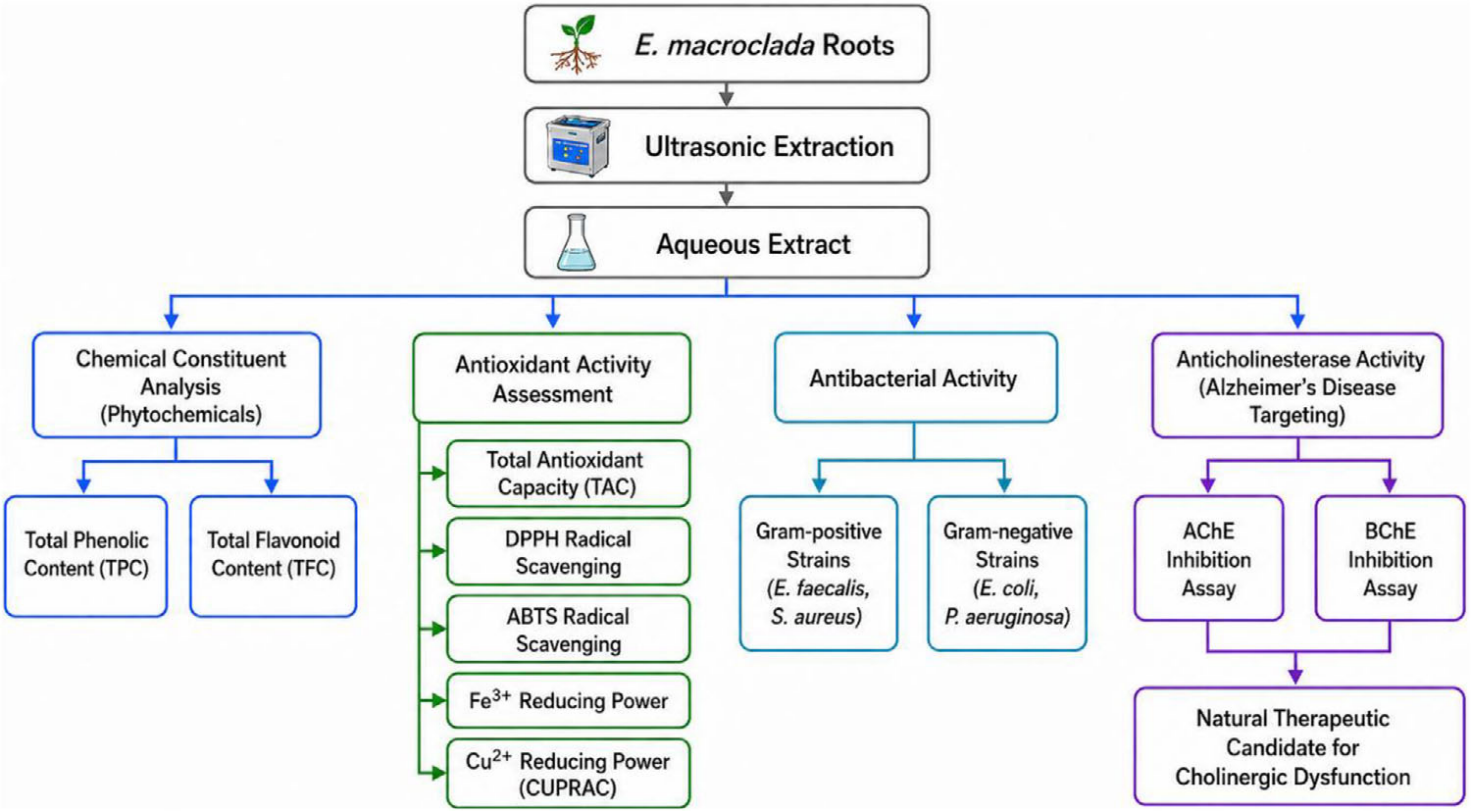
Schematic representation of the preparation of the extract from *E. macroclada* roots using the UAE method and the subsequent biological activity assays.

Plant extraction was carried out using the UAE method. The rationale for selecting UAE was to enhance extraction efficiency through the transmission of high‐frequency sound waves into a water bath containing the solvent and plant material. Acoustic cavitation facilitates the disruption of plant cell walls and increases the contact surface area between the solvent and bioactive compounds. This process improves both extraction rate and yield while enabling the preservation of thermolabile compounds [35]. For the UAE procedure, 5.0 g of powdered plant material was mixed with 100 mL of distilled water. The extraction was performed in an ultrasonic bath (WUC‐D10H, South Korea) operating at a constant power of 320 W and a frequency of 35 kHz for 120 min under laboratory conditions [36]. No additional heating was applied in order to preserve the active constituents of the plant material. At the end of the extraction period, the mixture was filtered. The filtrates were centrifuged at 10 000 rpm for 10 min using a centrifuge (Allegra X‐30R, Germany) to ensure purity. The solvent of the resulting extract was removed under reduced pressure at 50°C and 10 mbar using a rotary evaporator (Hei‐VAP Advantage, Heidolph, Germany) to obtain a dry powder. The extraction yield on a weight basis (*w/w*) was calculated using Equation (1). The ultrasonic extract was designated as Ult‐ER and stored at 4°C for further analyses.

(1) $$\mathrm{Yield}=\left(\frac{A_{1}}{A_{2}}\right)\times 100,$$

here, *A*_1_ represents the weight of the dried extract (*g*), and *A*_2_ denotes the weight of the plant powder (*g*) [23].

### 2.3 Determination of TPC and TFC in the Ult‐ER Extract

The TPC of the Ult‐ER extract (1 mg/mL) was determined using a colorimetric method based on the Folin–Ciocalteu reagent [37]. Briefly, 300 µL of the sample solution was diluted with 3.4 mL of deionized water, followed by the addition of 0.5 mL of methanol and 200 µL of Folin–Ciocalteu reagent. After vortexing, the mixture was incubated at room temperature for 10 min; subsequently, 600 µL of 10% (*w/v*) sodium carbonate (Na_2_CO_3_) solution was added, and the mixture was incubated in the dark for 120 min. At the end of the incubation period, absorbance was measured at 760 nm using a UV–vis spectrophotometer (UV‐1800, Shimadzu, Japan). A mixture containing deionized water instead of the sample served as the blank. TPC was calculated using a calibration curve constructed with gallic acid (GA) standards (25–250 µg/mL) (Figure 2A), and the results were expressed as mg gallic acid equivalents per gram of sample (mg GAE/g).

**FIGURE 2:**
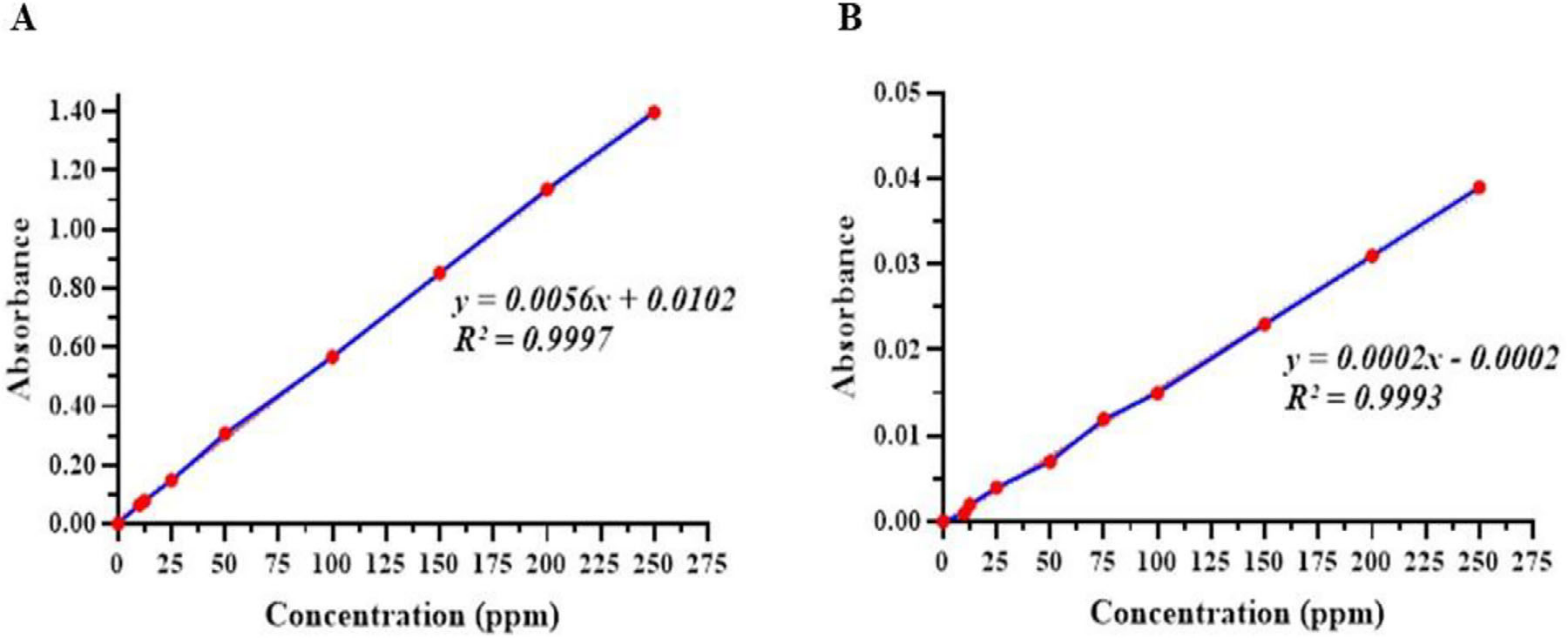
The standard curves for (A) TPC (*R* ^2^: 0.9997) and (B) TFC (*R* ^2^: 0.9993).

The TFC of the same root extract was determined using a colorimetric method based on complex formation with aluminum chloride (AlCl_3_) [37]. In brief, 500 µL of the extract was mixed with 3.2 mL of 30% methanol, followed by the addition of 150 µL of 5% (*w/v*) sodium nitrite (NaNO_2_), and the mixture was allowed to stand at room temperature for 5 min. Subsequently, 150 µL of 10% (*w/v*) AlCl_3_ and 1.0 mL of 1% (*w/v*) sodium hydroxide (NaOH) were added and thoroughly mixed. The absorbance of the reaction mixture was measured at 506 nm, using deionized water as the blank. TFC was calculated from a calibration curve prepared with quercetin (*Q*) standard solutions (25–250 µg/mL) (Figure 2B), and the results were expressed as mg quercetin equivalents per gram of sample (mg QE/g).

### 2.4 Determination of TAC of Ult‐ER by the Phosphomolybdenum Method

The TAC of the Ult‐ER extract was determined using a spectrophotometric method based on the phosphomolybdenum complex [38]. For this purpose, the phosphomolybdenum reagent was prepared from monobasic sodium phosphate, ammonium molybdate, and sulfuric acid, diluted to a final volume of 50 mL with methanol, and protected from light with aluminum foil due to its photosensitivity. In the assay, 500 µL of the extract was mixed with 2.5 mL of deionized water, followed by the addition of 1.0 mL of the phosphomolybdenum reagent. The mixture was incubated in a water bath at 95°C for 90 min and then allowed to cool at room temperature for 20–30 min. Standard antioxidant solutions, Trolox (T) and butylated hydroxyanisole (BHA), at concentrations of 25–250 µg/mL were subjected to the same procedure. The absorbance of the reaction products was measured at 695 nm against a blank containing 500 µL of deionized water. TAC values were calculated using calibration curves constructed with T and BHA standards and expressed as mg Trolox equivalents per gram (mg TE/g) and mg BHA equivalents per gram (mg BHAE/g) (Figure 3A,B).

**FIGURE 3:**
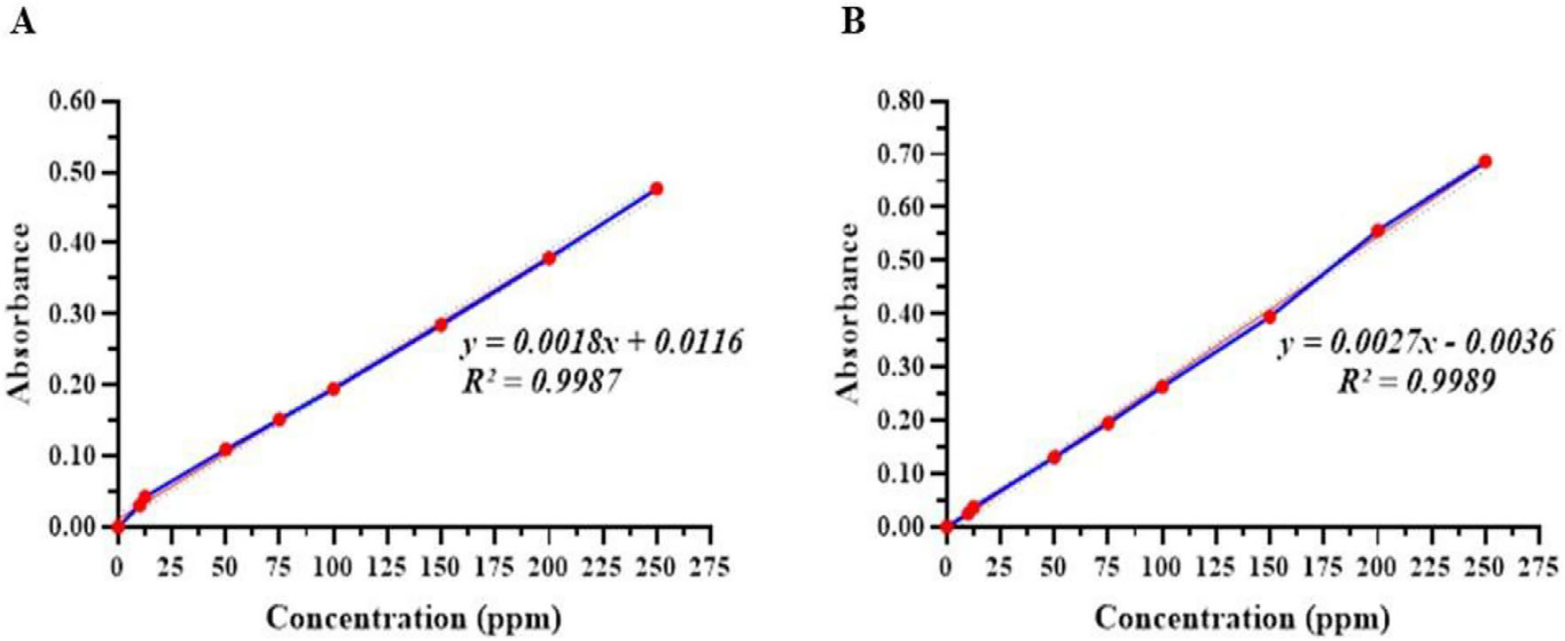
The standard curves for (A) T (*R* ^2^ = 0.9987) and (B) BHA (*R* ^2^ = 0.9989) for the TAC.

### 2.5 DPPH Free Radical Scavenging Activity of Ult‐ER

The DPPH free radical scavenging activity of the Ult‐ER extract was evaluated based on the protocol of Ahmed et al. [39] with minor modifications. A stock solution was prepared by dissolving 39.5 mg of DPPH in 10 mL of methanol and subsequently diluted with methanol to obtain a working solution with an absorbance of approximately 0.98 ± 0.02 at 517 nm. For the assay, 100 µL of the extract was mixed with 3.0 mL of the DPPH solution, and the mixtures were incubated in the dark at room temperature for 30 min. After incubation, absorbance values were recorded at 517 nm. Methanol (100 µL) was used instead of the sample as the blank. The same procedure was applied to T, BHA, and α‐tocopherol (α‐T) standards to generate calibration curves (Figure 4A–C). All analyses were performed in triplicate, and DPPH radical scavenging capacity was expressed as mg TE/L, mg BHAE/L, and mg α‐TE/L. The percentage inhibition was calculated according to Equation (2) [40].

(2) $$\mathrm{DPPH}\;\mathrm{free}\;\mathrm{radical}\;\mathrm{scavenging}\;\mathrm{activity}\;\left(\%\right)=\left(\frac{A_{0}-A_{1}}{A_{0}}\right)\times 100\;,$$

here, *A*_0_ represents the absorbance of the control containing all reagents except the test compounds, whereas *A*_1_ denotes the absorbance of the samples.

**FIGURE 4:**
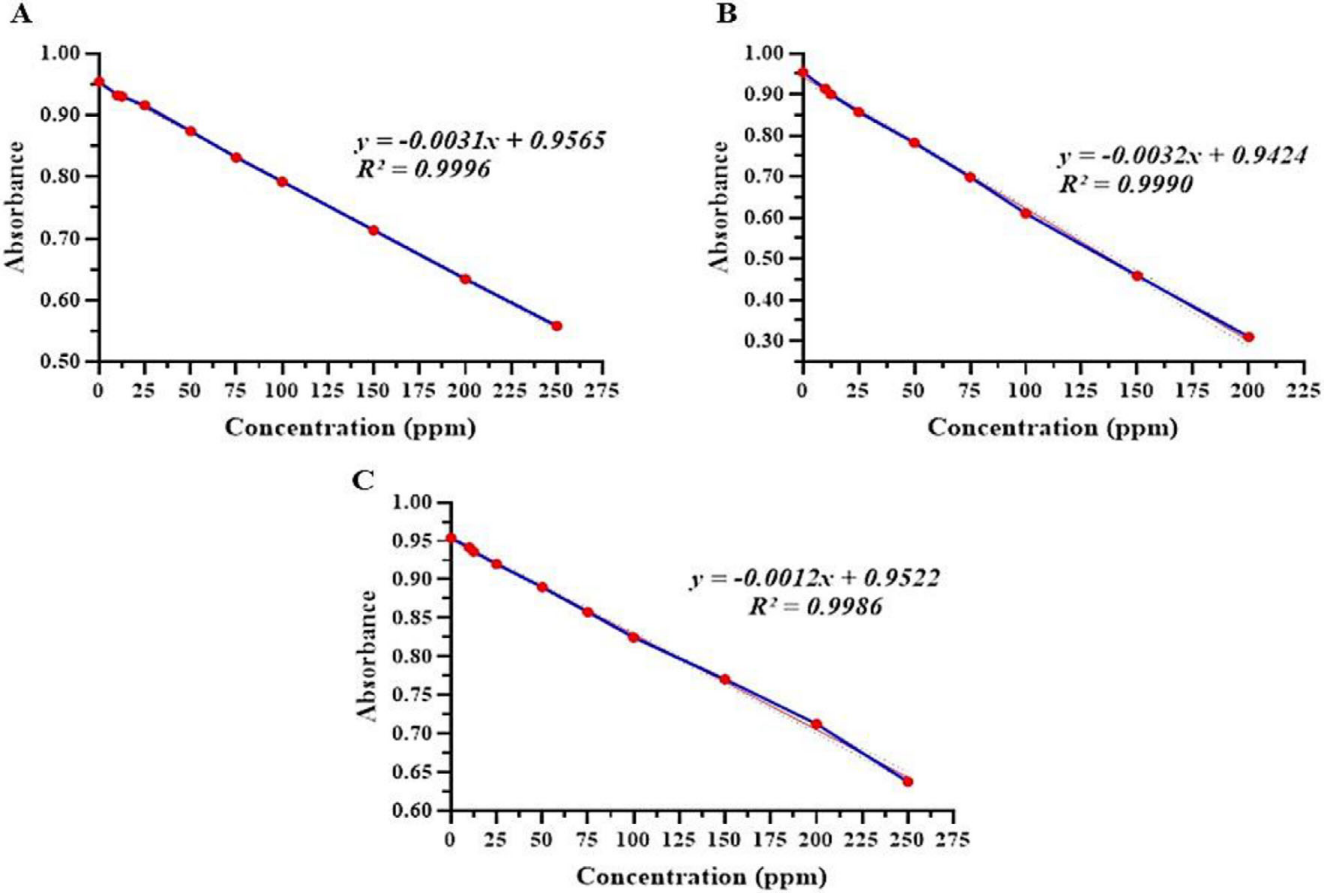
The standard curves for (A) T (*R* ^2^ = 0.9996), (B) BHA (*R* ^2^ = 0.9990), and (C) α‐Tocopherol (*R* ^2^ = 0.9986) for the DPPH.

### 2.6 ABTS^+^ Radical Scavenging Activity of Ult‐ER

The ABTS^+^ radical scavenging activity of the Ult‐ER extract was analyzed using a protocol adapted from Ahmed et al. [39]. A stock solution was prepared by mixing 7 mM ABTS with 2.45 mM potassium persulfate (K_2_S_2_O_8_) and allowing the mixture to stand in the dark for 12–16 h to generate the ABTS^+^ radical. The solution was then diluted with methanol to obtain an absorbance of 0.70 ± 0.02 at 734 nm. During the assay, 150 µL of the sample was mixed with 2.85 mL of the ABTS^+^ solution and incubated at room temperature for 10 min. Absorbance values were recorded at 734 nm. For the blank measurement, 150 µL of methanol was used instead of the sample. The same procedure was applied to T, BHA, and α‐T standards to construct calibration curves (Figure 5A–C). All analyses were performed in triplicate, and the results were expressed as mg TE/L, mg BHAE/L, and mg α‐TE/L. The percentage of radical inhibition was calculated using Equation (2).

**FIGURE 5:**
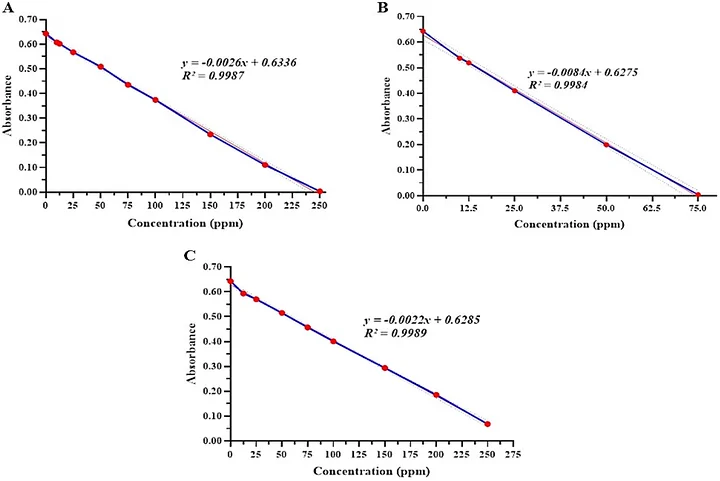
The standard curves for (A) T (*R* ^2^ = 0.9987), (B) BHA (*R* ^2^ = 0.9984), and (C) α‐Tocopherol (*R* ^2^ = 0.9989) for the ABTS^+^.

### 2.7 Fe^3+^─Fe^2+^ Reduction Capacity of Ult‐ER

The Fe^3+^ reducing power of the Ult‐ER extract was determined spectrophotometrically using a modified version of the method described by Oyaizu [41]. Briefly, 500 µL of the extract was mixed with equal volumes of 0.2 M phosphate buffer (PBS, pH 6.6) and 1% (*w/v*) potassium ferricyanide [K_3_Fe(CN)_6_]. After thorough mixing, the reaction mixture was incubated at 50°C for 20 min. The reaction was terminated by the addition of 10% (*w/v*) trichloroacetic acid (TCA), followed by brief centrifugation. Subsequently, 0.5 mL of the supernatant was mixed with 0.1% (*w/v*) ferric chloride (FeCl_3_) solution and allowed to stand at room temperature for 10 min. The absorbance of the resulting Prussian blue complex was measured at 700 nm against a phosphate buffer blank. The same procedure was applied to T and BHA standard solutions to construct calibration curves (Figure 6A,B). All analyses were performed in triplicate, and the reducing power was expressed as mg TE/L and mg BHAE/L.

**FIGURE 6:**
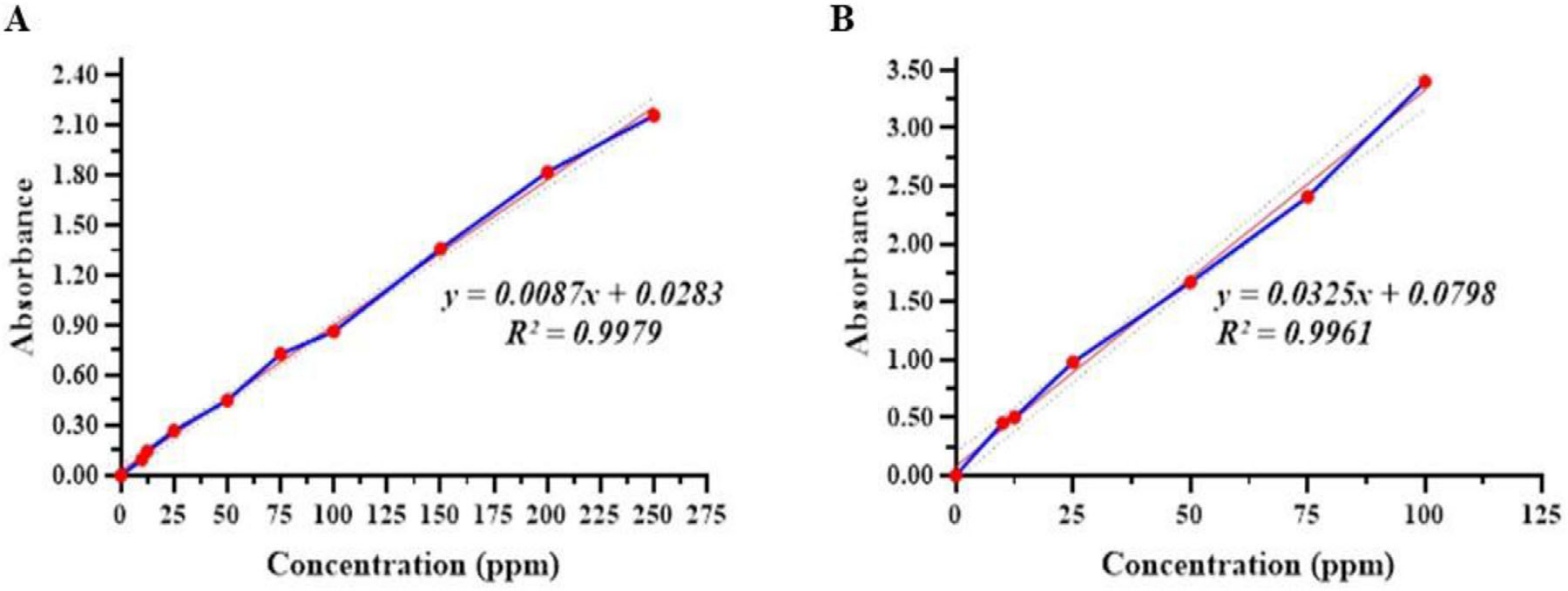
The standard curves for (A) T (*R* ^2^ = 0.9979) and (B) BHA (*R* ^2^ = 0.9961) for the Fe^3+^ reduction.

### 2.8 Cu^2+^─Cu^+^ Reduction Capacity of Ult‐ER

The electron transfer‐based antioxidant capacity of the Ult‐ER extract was determined using the method described by Apak et al.  [42], based on the reduction of Cu^2+^ to Cu^+^ in the presence of neocuproine. In brief, 1.0 mL of 0.01 M copper(II) chloride (CuCl_2_), 1.0 mL of 0.0075 M neocuproine, and 1.0 mL of ammonium acetate buffer (pH 7.0) were mixed with 0.5 mL of the sample solution. The total reaction volume was adjusted to 4.0 mL with deionized water. The reaction mixture was incubated in the dark at room temperature for 30 min, and the absorbance was measured at 450 nm against an acetate buffer blank. The same procedure was applied to T and BHA standard solutions to construct calibration curves (Figure 7A,B). The Cu^2+^ reducing capacity was calculated in triplicate, and the results were expressed as mg TE/L and mg BHAE/L.

**FIGURE 7:**
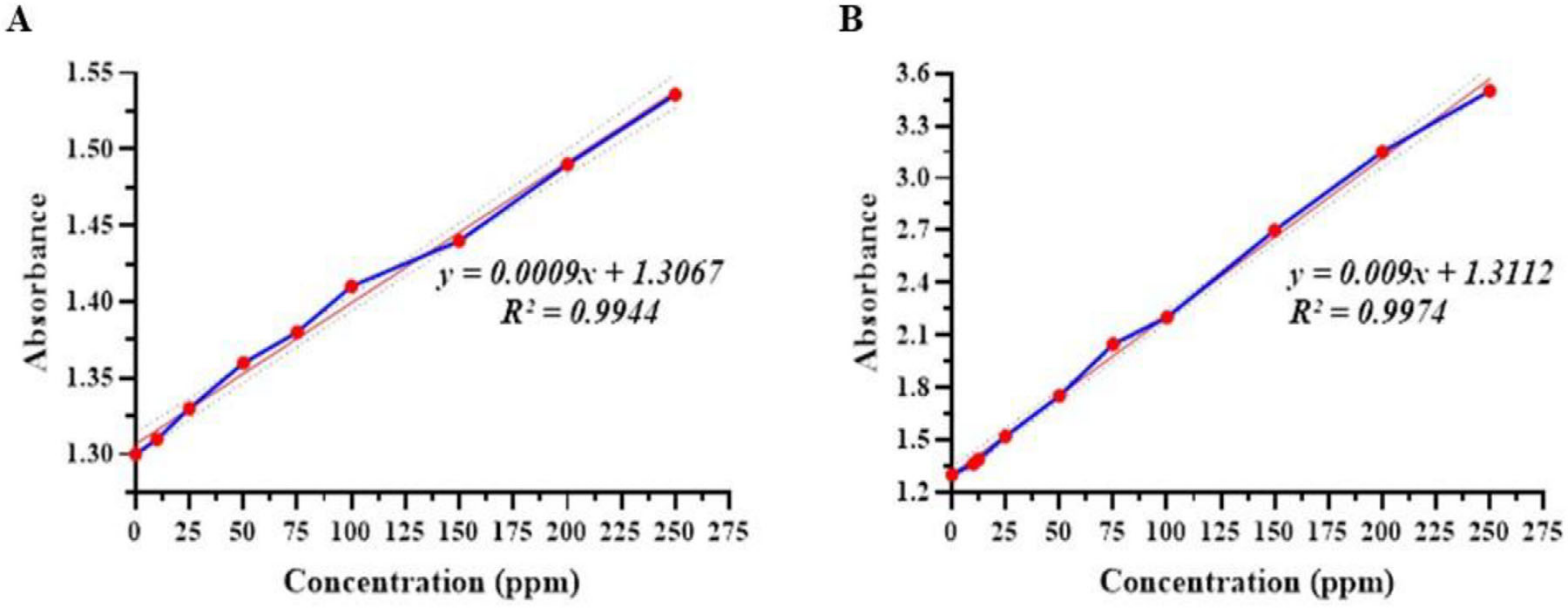
The standard curves for (A) T (*R* ^2^ = 0.9944) and (B) BHA (*R* ^2^ = 0.9974) for the Cu^2+^ reduction.

### 2.9 Antibacterial Activity of Ult‐ER

#### 2.9.1 Test Microorganisms

Nutrient broth (NB, 1.05443) and nutrient agar (NA, 1.05450) were used as culture media for the antibacterial assays, and ampicillin (A9393, ≥98%) was obtained from Merck KGaA (Germany). The bacterial strains employed in the antibacterial activity tests, *E. coli* ATCC 25922, *P. aeruginosa* ATCC 27853, *E. faecalis* ATCC 29212, and *S. aureus* ATCC 25923, were supplied by the Microbiology Laboratory of the Department of Food Engineering, Gümüşhane University.

#### 2.9.2 Agar Well Diffusion Method

The antibacterial activity of the Ult‐ER sample was evaluated against two Gram‐negative (*E. coli* and *P. aeruginosa*) and two Gram‐positive (*E. faecalis* and *S. aureus*) human pathogenic strains using the agar well diffusion method [43]. Initially, bacterial strains were reactivated in NB and incubated for 16–18 h to reach the logarithmic growth phase. Subsequently, the actively growing cultures were inoculated onto NA plates using the spread plate technique and allowed to adhere to the medium for 1 h. Wells of 6 mm diameter were then punched into the NA plates. Samples prepared in deionized water at different concentrations (Ult‐ER: 8000, 4000, 2000, and 1000 µg/mL) were added to the wells (50 µL per well) and allowed to diffuse at 25°C for 1 h. After diffusion, the plates were incubated at 37°C for 18 h. Inhibition zone diameters were measured in millimeters (mm) using a digital caliper. Ampicillin at a concentration of 25 µg/mL was used as the positive control.

### 2.10 AChE Inhibitory Activity of the Ult‐ER Extract

The AChE inhibitory capacity of the Ult‐ER extract was determined in vitro using a modified version of the colorimetric method described by Ellman et al.  [44]. In the experimental procedure, 10 µL of AChE was added to a mixture containing the inhibitor and 100 µL of buffer solution (1.0 M Tris/HCl, pH 8.0). The prepared mixture was incubated at 37°C for 10 min. Subsequently, the enzymatic reaction was initiated by adding 50 µL of 5,5′‐dithiobis‐2‐nitrobenzoate (DTNB, 10 mM) and 50 µL of the substrate (acetylthiocholine iodide). The thiocholine formed as a result of acetylthiocholine iodide hydrolysis by AChE reacted with DTNB to produce the yellow‐colored 5‐thio‐2‐nitrobenzoic acid, and the formation of this product was measured spectrophotometrically at 412 nm [45]. A control was prepared by adding an equal volume of deionized water instead of the sample. Tacrine at a concentration of 1 mg/mL was used as the positive control. All experiments were performed in triplicate, and the inhibitory activity of the Ult‐ER extract against AChE was evaluated based on the decrease in color formation, and IC_50_ (mg/mL) values were calculated.

### 2.11 BChE Inhibitory Activity of the Ult‐ER Extract

The BChE inhibitory potential of the Ult‐ER extract was determined in vitro using a modified version of the colorimetric method described by Ellman et al.  [44]. In the experimental procedure, 10 µL of BChE was added to a mixture containing the inhibitor and 100 µL of buffer solution (1.0 M Tris/HCl, pH 8.0). The prepared mixture was incubated at 37°C for 10 min. Subsequently, the enzymatic reaction was initiated by adding 50 µL of DTNB (10 mM) and 50 µL of the substrate (butyrylthiocholine iodide). BChE catalyzes the hydrolysis of butyrylthiocholine iodide to produce thiocholine and butyrate. The released thiocholine reacts with DTNB to form the yellow‐colored 5‐thio‐2‐nitrobenzoic acid, and the formation of this product was measured spectrophotometrically at 412 nm [46]. A control was prepared by adding an equal volume of deionized water instead of the sample. Tacrine at a concentration of 1 mg/mL was used as the positive control. All experiments were performed in triplicate, and the inhibitory activity of the Ult‐ER extract against BChE was evaluated based on the decrease in color formation, with IC_50_ (mg/mL) values calculated accordingly.

### 2.12 Statistical Analysis

All data obtained from antioxidant, anticholinesterase, and antibacterial assays were expressed as mean ± standard deviation (SD) of three technical replicates. Where applicable, statistical analyses were performed using one‐way analysis of variance (one‐way ANOVA) followed by Tukey's multiple‐comparisons test. Graphical and statistical evaluations were carried out using GraphPad Prism software version 10.0.1 and Microsoft Excel 2010. Differences were regarded as statistically significant when the *p*‐value was below 0.05. Statistical significance was indicated by asterisks as follows: *p* < 0.05 (^*^), *p* < 0.01 (^**^), *p* < 0.001 (^***^), and *p* < 0.0001 (^****^).

## 3 Results and Discussions

### 3.1 Bioactive Profile of Ult‐ER Extract

The TPC and TFC in plant extracts are important indicators reflecting the abundance of biologically active phytochemicals. Phenolic compounds possess redox properties due to their hydroxyl groups, enabling them to neutralize free radicals, and are therefore responsible for a substantial portion of antioxidant activity. Accordingly, a high TPC is generally considered an indicator of strong antioxidant potential in plant extracts. Similarly, flavonoids, which belong to the class of phenolic compounds, are associated with antioxidant, anti‐inflammatory, and other biological activities, and increases in TFC have been correlated with enhanced antioxidant capacity of the sample [47]. The phosphomolybdenum assay, used for TAC determination, is a widely applied method for evaluating the overall antioxidant potential of a sample. This assay is based on the formation of a phosphomolybdenum complex, allowing quantitative assessment of antioxidant capacity [48]. In light of this background, the yield, TPC, TFC, and TAC values of the aqueous extract obtained from the roots of *E. macroclada* by UAE Ult‐ER were determined, and the results are presented in Table 1.

**TABLE 1:** Levels of TPC, TFC, and TAC in the Ult‐ER extract^a^.

Sample	TAC		TPC (mg GAE/g)	TFC (mg QE/g)	Yield (%)
	(mg TE/g)	(mg BHAE/g)			
**Ult‐ER**	9.78 ± 0.44	15.62 ± 1.85	84.80 ± 3.31	60.67 ± 2.29	10.50

^a^ Values are expressed as mean ± SD of three technical replicates (*n* = 3).

When the extraction yield of the Ult‐ER extract was evaluated, the 10.50% yield may be associated with the high lignocellulosic content of the root tissues and the fact that a considerable proportion of phenolic compounds are present in cell wall‐bound forms. It is well established that the cavitation effect generated during UAE disrupts cell wall integrity, enhances solvent penetration, and significantly improves extraction efficiency compared with conventional maceration methods [49]. Although, to the best of our knowledge, no study has reported the extraction yield of *E. macroclada* roots obtained by UAE, several investigations have been conducted on different species within the genus *Euphorbia*. A review of studies performed on various *Euphorbia* species using different extraction approaches indicates that extraction yields vary widely depending on plant species, solvent type, plant part, and extraction technique. For instance, in aqueous and ethanolic extracts obtained from different morphological parts of *Euphorbia characias*, the highest yields were reported in ethanol extracts, particularly from the leaves (24.9%), while aqueous extract yields ranged between 12.3% and 20.4% [23]. In *Euphorbia nivulia*, alcoholic extracts and fractions obtained from aerial parts by maceration revealed a remarkably high yield for the aqueous fraction (84%), whereas the crude extract yield was limited to 7.14% [50]. This finding highlights the decisive role of fractionation and solvent polarity in extraction efficiency.

Similarly, in a study on *Euphorbia milii* where roots and aerial parts were extracted by maceration using dichloromethane and methanol, root extract yields ranged between 11% and 19%, with methanol providing higher yields in root tissues [51]. In *E. gaillardotii* and *E. macroclada*, maceration extracts obtained using petroleum ether and methanol exhibited yields ranging from 2.17% to 9.48% [52]. Furthermore, methanolic extracts of *E. macroclada* roots collected from different regions of Türkiye and obtained by maceration showed yields between 5.69% and 14.79%, with variations attributed to geographical origin, harvesting time, and morphological characteristics of the plant [53]. In this context, the 10.50% extraction yield determined for the aqueous Ult‐ER sample obtained from *E. macroclada* roots by UAE is consistent with and noteworthy compared with the maceration‐based studies reported in the literature. Considering that UAE enables efficient extraction within a shorter time and with lower energy and solvent consumption, the yield obtained in the present study can be regarded as both methodologically advantageous and sufficient for subsequent biological and functional analyses. Therefore, this study demonstrates that aqueous extracts of *E. macroclada* roots can be effectively obtained using UAE, contributing to sustainable and green extraction strategies within the genus *Euphorbia*.

The findings presented in Table 1 indicate that the Ult‐ER sample contains phenolic and flavonoid constituents at appreciable levels. Specifically, the TPC was 84.8 ± 3.31 mg GAE/g, whereas the TFC was 60.67 ± 2.29 mg QE/g. Phenolic compounds are known to play a central role in the antioxidant potential of plant extracts, exerting their effects through mechanisms such as free‐radical scavenging, metal‐ion chelation, and suppression of oxidative chain reactions [54]. In this context, the elevated TPC and TFC values determined in the Ult‐ER sample suggest that the extract possesses strong biological potential. Flavonoids, which constitute a broad class of polyphenolic compounds, exhibit various biological functions including antiulcer, antiangiogenic, anticancer, and antiarthritic activities, as well as the inhibition of protein kinase activity [47]. These compounds, characterized by a benzo‐γ‐pyrone skeleton, have also been reported to display significant antimicrobial properties [55]. Moreover, flavonoids contribute to antioxidant defense by scavenging ROS, particularly superoxide and hydroxyl radicals, thereby playing a protective role against cardiovascular diseases, certain types of cancer, and age‐related cellular damage [56].

When compared with previously reported studies on *Euphorbia* species, the present findings place the TPC and TFC values of Ult‐ER within the range reported for different members of the genus. For instance, TPC values ranging from 35.84 to 141.90 mg GAE/g have been reported in methanolic and aqueous extracts of three different *Euphorbia* species [57]. Accordingly, the TPC of Ult‐ER (84.8 ± 3.31 mg GAE/g) falls within this previously reported range. Similarly, in aqueous and ethanolic extracts obtained from different morphological parts of *E. characias*, the highest TPC and TFC values were reported to be approximately 800 mg GAE/g and 241.7 mg QE/g, respectively [23]. More specifically, the TPC and TFC values of Ult‐ER were numerically higher than the corresponding values of 53.73 ± 0.30 mg GAE/g and 44.62 ± 0.38 mg QE/g reported for the methanolic extract of *E. parviflora* [58]. Organ‐specific TPC values of 206.17 ± 1.95, 117.08 ± 3.10, 83.15 ± 1.19, and 65.70 ± 1.72 mg GAE/g have also been reported for the leaf, flower, root, and stem extracts of *Euphorbia hirta*, respectively [59]. Notably, the TPC of Ult‐ER was comparable to that of the *E. hirta* root extract and higher than that of the stem extract. In another study, an alcoholic root extract of *E. hirta* exhibited a higher TPC value of 265.72 ± 1.30 mg GAE/g but a lower TFC value of 45.67 ± 1.14 mg QE/g compared with Ult‐ER [60]. The TPC of Ult‐ER was also higher than those reported for the aqueous and methanolic extracts of *E. tirucalli* (34.01 and 77.33 mg GAE/g, respectively) [61]. Differences between the present findings and previously reported values may be associated with plant species and chemotype, the examined plant organ, solvent polarity, extraction conditions, phenological stage, growing environment, and the basis used to express the results. Collectively, these comparisons show that the TPC and TFC values of Ult‐ER are comparable to or higher than those of several previously studied *Euphorbia* extracts, highlighting *E. macroclada* roots as a relevant source of phenolic and flavonoid constituents. In the TAC analysis, values of 9.78 ± 0.44 mg TE/g and 15.62 ± 1.85 mg BHAE/g were obtained for the Ult‐ER sample. In a study conducted by Saleem et al.  [51], the TAC values of methanol and DCM extracts prepared from the aerial and root parts of *E. milii* were reported to range between 0.38 and 1.91 mg TE/g. Compared with that study, the present work yielded higher TAC values.

While TPC and TFC analyses reflect the total amounts of phenolic and flavonoid compounds present in the extract, TAC analysis represents a functional response based on the overall electron‐donating and reducing capacity of the extract. The antioxidant activity of phenolic compounds depends not only on their concentration but also on their molecular structure, number and position of hydroxyl groups, and degree of conjugation [54, 62]. Therefore, extracts with high TPC and TFC contents do not always exhibit proportionally high antioxidant capacity, as widely reported in the literature [63]. Indeed, several studies have demonstrated non‐linear or weak correlations between TPC and TFC values and TAC results [64]. This indicates that antioxidant activity is strongly influenced not only by the quantity of compounds but also by the qualitative characteristics of the phenolic profile. The concurrent detection of TPC, TFC, and TAC responses suggests that the phenolic and flavonoid constituents of Ult‐ER may contribute to its overall reducing capacity. Numerous studies have emphasized a strong positive correlation between phenolic content and TAC [40]. The UAE method employed in the present study has been reported to disrupt cell wall integrity, thereby facilitating the transfer of phenolic compounds into the solvent phase and yielding higher levels of bioactive compounds compared to conventional extraction techniques [65]. This may represent one of the underlying reasons for the observed TPC and TFC values obtained in the Ult‐ER sample. The Ult‐ER extract obtained from *E. macroclada* roots demonstrated appreciable phenolic and flavonoid contents together with measurable total antioxidant capacity. These findings support the further investigation of *E. macroclada* roots as a potential source of antioxidant constituents. Accordingly, further studies focusing on the isolation of the compounds responsible for this activity and clarification of their underlying mechanisms would provide valuable insight.

### 3.2 Antioxidant Radical Scavenging (DPPH/ ABTS^+^) by Ult‐ER Extract

Antioxidants play a protective role in both food systems and biological organisms by limiting oxidative damage caused by ROS. In addition to reducing protein and amino acid oxidation, these compounds contribute to the preservation of biomolecular integrity by restricting the interaction of lipid‐derived reactive carbonyl compounds with proteins [66]. Plants produce a wide variety of secondary metabolites, including phenolic acids, flavonoids, terpenoids, and tocopherols, which function as natural antioxidants [62]. To determine the antioxidant capacity of plant extracts and other biological samples, DPPH and ABTS^+^ radical scavenging assays are widely accepted as simple, sensitive, and reproducible in vitro methods that evaluate the ability of different antioxidant compounds to neutralize free radicals. The DPPH method is based on the reduction of the DPPH radical by antioxidants, resulting in a decrease in absorbance that reflects the radical scavenging capacity of the sample. The ABTS^+^ radical scavenging assay measures the ability to neutralize the color of the ABTS^+^ radical cation, which is applicable in both aqueous and organic solvent systems, thereby providing a suitable antioxidant assessment across a broad range of compounds [67].

In the present study, the antioxidant potential of Ult‐ER obtained from the roots of *E. macroclada* by UAE was evaluated using DPPH and ABTS^+^ radical scavenging assays (Figure 8). The simultaneous use of T, BHA, and *α*‐T standards in both DPPH and ABTS^+^ assays enables comparison of the extract's antioxidant capacity according to different redox mechanisms. T, a water‐soluble analog of vitamin E, is widely accepted as a universal reference standard reflecting the electron transfer capacity of hydrophilic antioxidants [68]. BHA, due to its strong synthetic phenolic structure, is commonly employed for comparing natural extracts with industrial antioxidants [69]. In contrast, α‐T is one of the principal lipophilic antioxidants that prevents lipid peroxidation in biological systems and allows assessment of the potential efficacy of plant extracts under physiological conditions [70]. This multi‐standard approach enables a comprehensive functional interpretation of antioxidant capacity rather than a one‐dimensional evaluation.

**FIGURE 8:**
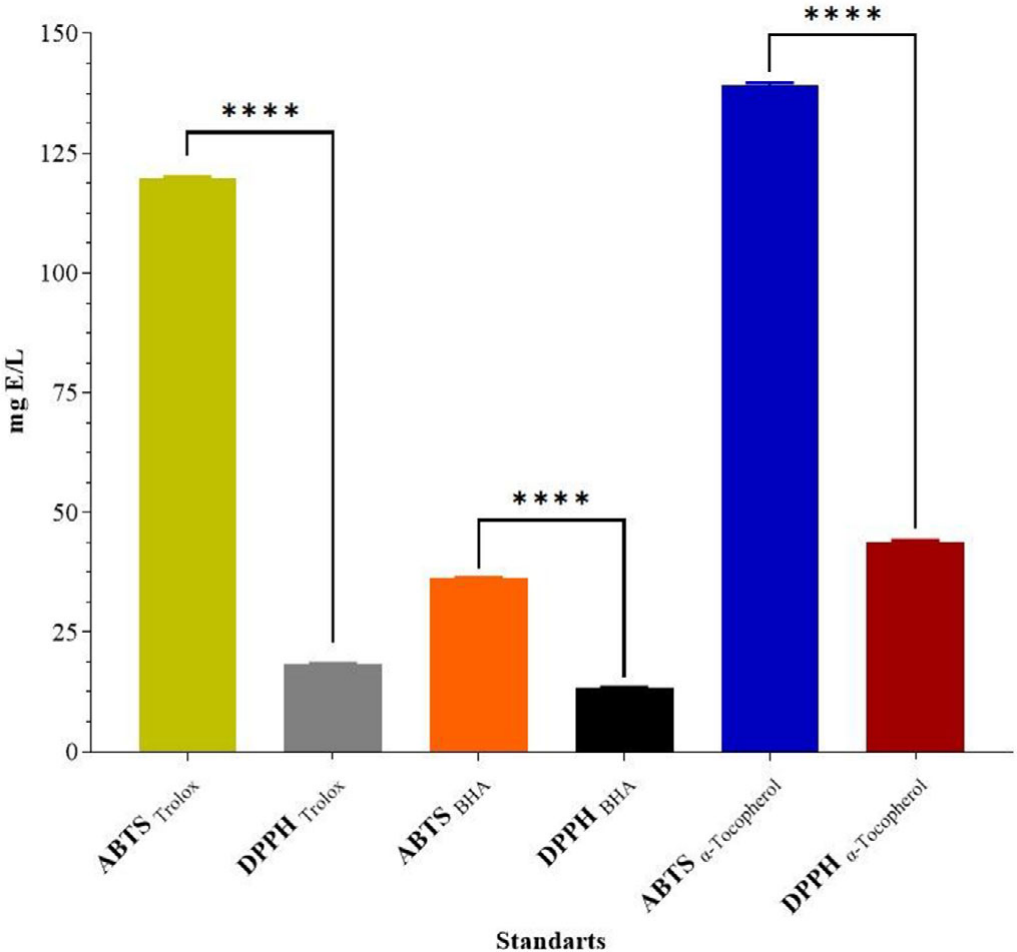
Comparative DPPH and ABTS^+^ radical scavenging capacities of Ult‐ER extract expressed as Trolox, BHA, and *α*‐Tocopherol equivalents. Data are presented as mean ± SD of three technical replicates (*n* = 3). Group‐wise comparisons were conducted using one‐way ANOVA with Tukey's post hoc test; ^****^ indicates *p* < 0.0001.

As shown in Figure 8, the antioxidant capacity of Ult‐ER in the DPPH free radical scavenging assay was determined as 18.33 ± 0.49 mg TE/L, 13.35 ± 0.80 mg BHAE/L, and 43.78 ± 1.20 mg α‐TE/L. Due to its lipophilic nature, the DPPH radical is particularly sensitive to low‐molecular‐weight phenolic compounds with high hydrogen‐donating capacity. Therefore, highly polar phenolic acids, flavonoid glycosides, and bound phenolic forms commonly present in aqueous extracts may exhibit limited reactivity toward the DPPH radical [64, 71]. This may represent a key factor explaining the comparatively moderate scavenging performance of Ult‐ER in the DPPH assay. In addition, the percentage inhibition of Ult‐ER was found to remain at a relatively low level (5.70% ± 0.06%).

As also illustrated in Figure 8, Ult‐ER exhibited a remarkably high antioxidant capacity in the ABTS^+^ radical scavenging assay. In this assay, values of 119.59 ± 1.86 mg TE/L, 36.29 ± 1.80 mg BHAE/L, and 139.02 ± 4.81 mg α‐TE/L. were obtained. The inhibition percentage was determined as 49.82% ± 0.024%. Since the ABTS^+^ radical is responsive to both hydrophilic and lipophilic antioxidants, it can more comprehensively reflect the overall redox capacity of aqueous extracts [72]. Therefore, the significantly higher activity of Ult‐ER in the ABTS^+^ assay compared to the DPPH assay is an anticipated outcome and is consistent with the existing literature.

As also illustrated in Figure 8, Ult‐ER exhibited a remarkably high antioxidant capacity in the ABTS^+^ radical scavenging assay. In this assay, values of 119.59 ± 1.86 mg TE/L, 36.29 ± 1.80 mg BHAE/L, and 139.02 ± 4.81 mg α‐TE/L were obtained. The inhibition percentage was determined as 49.82% ± 0.024%. Because the ABTS^+^ radical is responsive to both hydrophilic and lipophilic antioxidants, it can more comprehensively reflect the overall redox capacity of aqueous extracts [72]. Therefore, the significantly higher activity of Ult‐ER in the ABTS^+^ assay compared to the DPPH assay is an anticipated outcome and is consistent with the existing literature.

To date, the DPPH and ABTS^+^ radical scavenging activities of the extract obtained from *E. macroclada* roots using the UAE method have not been reported in the literature. However, studies conducted on different *Euphorbia* species and various plant parts indicate that antioxidant capacity varies depending on both the plant species and the extraction solvent employed. For instance, in a study by Saleem et al.  [51], the aerial parts and root extracts of *E. milii* were evaluated using DPPH and ABTS^+^ radical scavenging assays. The methanolic root extract exhibited the highest activity in both DPPH and ABTS^+^ assays (58.54 and 89.14 mg TE/g, respectively), whereas the dichloromethane extract of the aerial parts showed the lowest values (7.23 and 22.71 mg TE/g, respectively). In all samples, ABTS^+^ radical scavenging values were higher than those obtained from the DPPH assay. Similarly, another study examined the aqueous, methanolic, and ethyl acetate extracts of *E. hirta*, *E. heterophylla*, and *E. convolvuloides*. Among the three species, the methanolic extract of *E. hirta* demonstrated the strongest antioxidant activity in terms of DPPH (195.22 mg TE/g) and ABTS^+^ radical scavenging (324.28 mg TE/g) [57]. The study further reported that methanolic and aqueous extracts exhibited higher antioxidant capacities, whereas ethyl acetate extracts showed comparatively lower activity [57]. These findings indicate that hydrophilic antioxidant compounds play a prominent role in *Euphorbia* species and that both the assay type and solvent system substantially influence the measured antioxidant activity. In the present study, the Ult‐ER extract obtained from *E. macroclada* roots exhibited high antioxidant capacity values in both DPPH and ABTS^+^ radical scavenging assays. These results suggest that Ult‐ER is particularly rich in hydrophilic antioxidant compounds and demonstrates markedly higher antioxidant capacity compared with previously reported values for *Euphorbia* species. Collectively, the findings highlight the biological relevance of *E. macroclada* roots as a promising source of natural antioxidants and suggest their potential significance for future research on plant‐derived antioxidant systems.

### 3.3 Antioxidant Reducing Potential (Fe^3+/^Fe^2+^ and Cu^2+^/Cu^+^ Reduction) of Ult‐ER Extract

Reduction‐based assays (Fe^3+^/Fe^2+^ and Cu^2+^/Cu^+^ reduction systems) are widely employed for the determination of antioxidant capacity in a given sample. The Fe^3+^ reduction assay evaluates the ability of antioxidants to reduce ferric ions (Fe^3+^) to the ferrous form (Fe^2+^), thereby primarily reflecting the overall reducing power of hydrophilic antioxidants [73]. In contrast, the Cu^2+^ reduction method is based on the reduction of Cu^2+^ ions to Cu^+^ in the presence of neocuproine and enables the assessment of a broader antioxidant profile, as it is responsive to both hydrophilic and lipophilic antioxidants [74]. The combined application of these reduction‐based approaches allows complementary evaluation of antioxidants with different redox potentials, contributing to a more reliable interpretation of the total antioxidant capacity of the sample [75].

In the present study, the antioxidant potential of Ult‐ER obtained from *E. macroclada* roots via UAE was determined using Fe^3+^ and Cu^2+^ ion reduction assays (Figure 9). The results demonstrated that the Ult‐ER extract possesses a pronounced electron‐donating capacity. The Cu^2^^+^ reduction capacity of Ult‐ER was approximately 670.33± 11.59 mg TE/L, whereas its Fe^3^^+^ reduction capacity was approximately 128.51 ± 12.57 mg TE/L. A similar pattern was observed when the results were expressed as BHA equivalents, with values of approximately 66.53± 4.11 mg BHAE/L for Cu^2^^+^ reduction and 32.82 ± 3.37 mg BHAE/L for Fe^3^^+^ reduction. These results indicate that Ult‐ER exhibited metal‐ion‐reducing activity in both assays, with a higher response in the Cu^2^^+^‐based system under the tested conditions.

**FIGURE 9:**
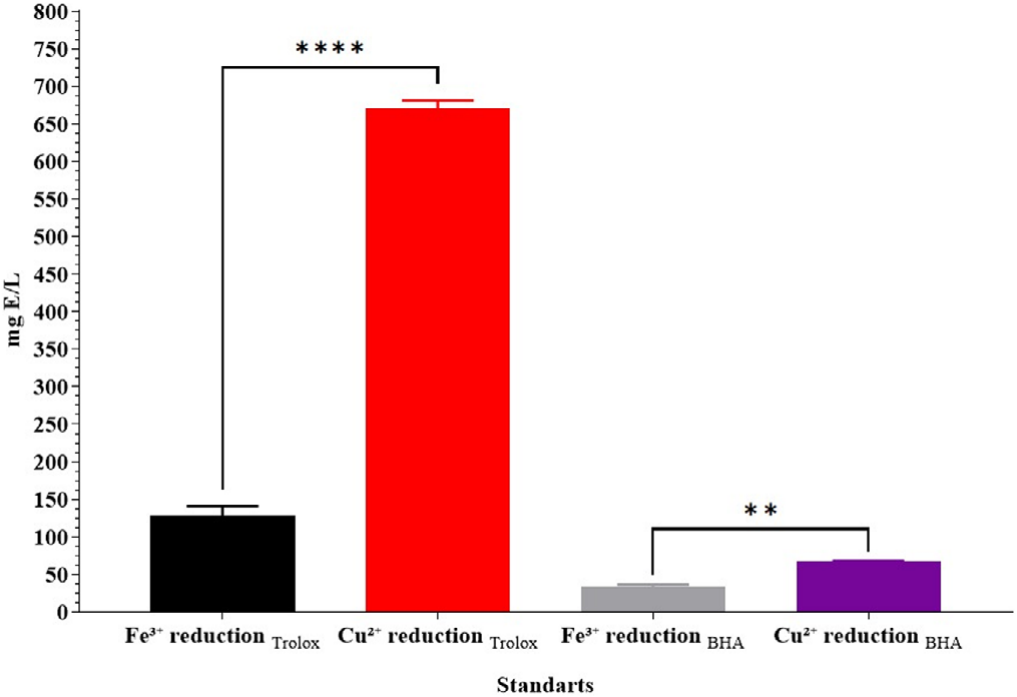
Comparative Fe^3+^ and Cu^2+^ reduction capacities of Ult‐ER extract expressed as Trolox, and BHA. Data are presented as mean ± SD of three technical replicates (*n* = 3). Group‐wise comparisons were conducted using one‐way ANOVA with Tukey's post hoc test; ^****^ indicates *p* < 0.0001.

According to the literature, the Fe^3+^ reduction assay is generally more sensitive to hydrophilic antioxidants, whereas the Cu^2+^ reduction method can detect both hydrophilic and lipophilic antioxidant compounds [74]. Therefore, the higher Cu^2+^ reduction capacity observed in this study may indicate the presence of phenolic acids, flavonoids, and other compounds possessing multiple redox‐active centers, such as ascorbic acid‐like molecules, in the Ult‐ER extract. Furthermore, the Cu^2+^/Cu^+^ redox couple has a lower redox potential compared to the Fe^3+^/Fe^2+^ system, which generally results in higher reduction responses of plant extracts in the Cu^2+^‐based assay [42, 63]. This mechanistic difference scientifically explains the significantly higher Cu^2+^ reduction values compared with the Fe^3+^ reduction results obtained in the present study.

Studies investigating the antioxidant potential of *Euphorbia* species have used reduction‐based assays to characterize the electron‐donating properties of extracts from this genus. In this context, FRAP analyses performed on aqueous and ethanolic extracts obtained from the leaves, stems, and flowers of *E. characias* revealed that the highest FRAP values were observed in ethanolic extracts, reported as 4.57, 1.36, and 4.49 mmol Fe^2^^+^/g, respectively. These findings indicate that reducing capacity varies considerably depending on both the plant organ and the extraction solvent [23]. In another study, the Fe^3^^+^ reducing potential of an extract obtained from the latex of *E. dendroides* L. was evaluated using the FRAP method, and the FRAP value was reported as 4383.13 µmol TE/100 g fresh weight. These results demonstrate that the Fe^3^^+^ reduction capacity of *Euphorbia* species can vary over a wide range depending on species and matrix characteristics [76]. Similarly, CUPRAC and FRAP assays conducted on aerial‐part and root extracts of *E. milii* showed that the methanolic root extract exhibited the highest reducing power, with FRAP and CUPRAC values of 87.73 and 136.44 mg TE/g, respectively [51]. In that study, CUPRAC values were higher than FRAP results, illustrating the assay‐dependent nature of the measured reducing response. A similar pattern was observed in the present study: the Cu^2+^ reduction capacity of Ult‐ER was 670.33 ± 11.59 mg TE/L, whereas its Fe^3+^ reduction capacity was 128.51 ± 12.57 mg TE/L. When expressed as BHA equivalents, the corresponding values were 66.53 ± 4.11 mg BHAE/L for Cu^2+^reduction and 32.82 ± 3.37 mg BHAE/L for Fe^3+^ reduction. Mahomoodally et al.  [57] compared the FRAP and CUPRAC values of water, methanol, and ethyl acetate extracts from *E. hirta*, *E. heterophylla*, and *E. convolvuloides* and reported that the aqueous extract of *E. heterophylla* exhibited the highest Fe^3^^+^ (451.08 mg TE/g) and Cu^2^^+^ (631.25 mg TE/g) reducing capacities. Since these literature values were expressed on a mass basis, whereas the present results were expressed on a volume basis, they provide useful contextual information but do not permit a direct quantitative ranking of reducing capacity. Differences in plant species, tissues, extraction conditions, and assay procedures should also be considered when interpreting these results.

Collectively, the results demonstrate that Ult‐ER exhibited measurable reducing activity in both metal‐ion‐based assays, with a higher response in the Cu^2+^ reduction system under the tested conditions. This assay‐dependent profile supports the in vitro electron‐donating potential of the extract, while further chemical profiling and complementary biological studies are required to identify the compounds responsible for this activity and clarify its biological relevance.

### 3.4 Antibacterial Analysis Results

Antimicrobial resistance is recognized among the top 10 global public health threats, causing approximately 700,000 deaths annually [77] and representing a serious concern for healthcare systems worldwide. This situation clearly underscores the urgent need for the development of new and effective antimicrobial agents. Natural sources, particularly plants, provide a vast array of bioactive compounds with diverse chemical structures and unique pharmacological mechanisms of action [78]. Therefore, the discovery of antimicrobial agents from natural resources has gained considerable importance. In this context, the present study evaluated the antibacterial activity of Ult‐ER extract prepared at different concentrations (8000, 4000, 2000, and 1000 µg/mL) against Gram‐positive bacteria (*S. aureus* and *E. faecalis*) and Gram‐negative bacteria (*E. coli* and *P. aeruginosa*). Antibacterial activity was determined using the agar well diffusion method, and ampicillin (25 µg/mL) was used as the positive control.

One of the primary factors influencing the antibacterial performance of the Ult‐ER sample is the applied concentration. Accordingly, testing the extract at different dose levels (8000, 4000, 2000, and 1000 µg/mL) is essential for elucidating the dose–response relationship. In medical applications or environmentally sensitive settings, identifying the lowest effective concentration not only offers economic advantages but also contributes to minimizing potential adverse effects. Conversely, higher concentrations facilitate the determination of maximal inhibition levels. Therefore, the simultaneous evaluation of multiple concentrations in this study was scientifically justified to determine the minimum effective dose, analyze dose dependency, and define optimal conditions. Antibacterial activity measurements were conducted at the specified concentrations, and the resulting inhibition zone diameters were calculated in mm and presented in Table 2. Representative Petri‐plate images obtained from the experimental procedures are shown in Figure 10.

**TABLE 2:** Antibacterial activity profile of the Ult‐ER extract against Gram‐positive and Gram‐negative bacteria.

	Diameter of inhibition zones, mm*			
	Gram‐negative		Gram‐positive	
Samples	*E. coli*	*P. aeruginosa*	*S. aureus*	*E. faecalis*
Ult‐ER1‐ 8 mg/mL	11.10 ± 0.7^b^	10.25 ± 0.6^b^	10.75 ± 0.7^b^	10.85 ± 0.7^b^
Ult‐ER2‐ 4 mg/mL	NA	NA	NA	NA
Ult‐ER3‐ 2 mg/mL	NA	NA	NA	NA
Ult‐ER4‐ 1 mg/mL	NA	NA	NA	NA
Ampicillin (25 µg/mL)	18.30 ± 1.0^a^	32.70 ± 1.5^a^	13.75 ± 0.8^a^	14.50 ± 0.9^a^

*The data are expressed as mean ± SD (*n* = 3). Different letters within the same column indicate significant differences according to Tukey's test (*p* < 0.05). NA: no detectable inhibition zone.

**FIGURE 10:**
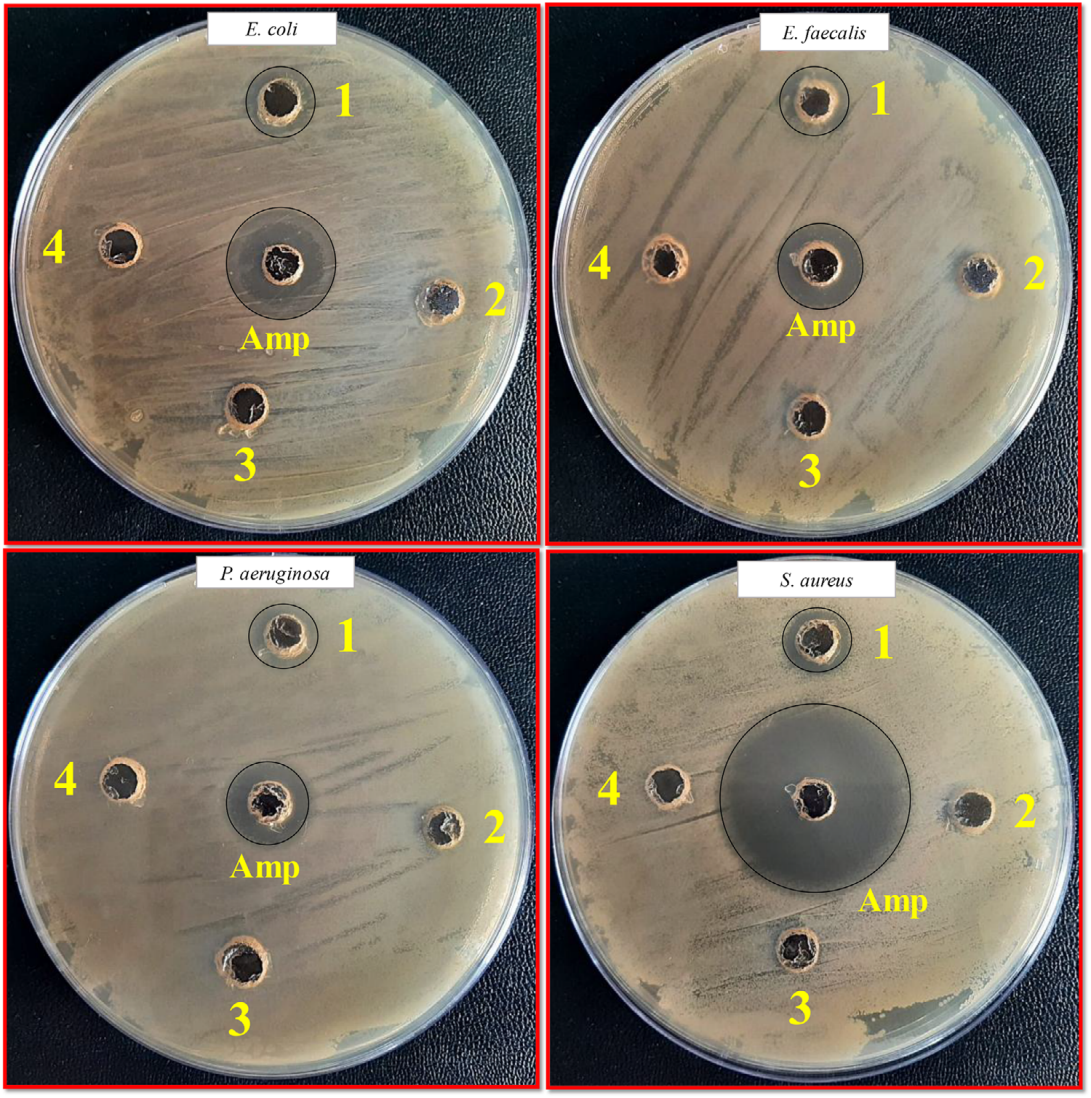
Representative images of Petri plates demonstrating antibacterial activity (1: Ult‐ER, 8 mg/mL; 2: Ult‐ER, 4 mg/mL; 3: Ult‐ER, 2 mg/mL; 4: Ult‐ER, 1 mg/mL; Amp: ampicillin, 25 µg/mL).

Figure 10 presents representative Petri plates illustrating the antibacterial effects of different concentrations of the Ult‐ER extract against *E. coli*, *P. aeruginosa*, *S. aureus*, and *E. faecalis*. As observed in the image, the 8 mg/mL Ult‐ER concentration (labeled as 1) produced distinct inhibition zones against all four bacterial strains. In contrast, no inhibition zones were detected at the lower concentrations (4, 2, and 1 mg/mL). These findings indicate that the antibacterial activity of the extract became detectable at the highest concentration evaluated, suggesting a concentration‐related response under the tested conditions. The broad and well‐defined inhibition zones observed in the ampicillin control confirm the proper performance of the positive control.

Evaluation of the data presented in Table 2 further demonstrates that the extract exhibited measurable antibacterial activity only at 8 mg/mL. At this concentration, inhibition zone diameters of 11.1 ± 0.7, 10.25 ± 0.6, 10.75 ± 0.7, and 10.85 ± 0.7 mm were recorded against *E. coli*, *P. aeruginosa*, *S. aureus*, and *E. faecalis*, respectively. No activity was detected at lower concentrations (1–4 mg/mL). As expected, ampicillin produced larger inhibition zones; nevertheless, Ult‐ER exhibited measurable activity against all four bacterial strains at 8 mg/mL. These results support the further evaluation of Ult‐ER as a potential source of antibacterial constituents. This observation is consistent with literature reports indicating that secondary metabolites such as phenolic compounds, flavonoids, diterpenoids, and triterpenoids may increase bacterial membrane permeability and disrupt enzymatic functions [79]. The comparable inhibition levels observed against the Gram‐positive and Gram‐negative bacteria suggest that the extract has antibacterial potential across the tested bacterial panel. The measurable inhibition zones produced by the crude extract suggest that its antibacterial activity may be further enhanced through purification or bioactivity‐guided fractionation. Previous studies have attributed the antibacterial activity of plant extracts to multiple mechanisms. Phenolic and terpenoid compounds may disrupt membrane stability, leading to leakage of intracellular contents—a mechanism frequently reported in studies on plant‐derived antimicrobial agents [80]. Additionally, plant extracts may induce oxidative stress in bacterial cells, resulting in lipid peroxidation and DNA damage [81].

The antibacterial activities of several *Euphorbia* species have been documented in the literature. For instance, extracts of *E. helioscopia* prepared at a concentration of 10 mg/mL were evaluated against MRSA (*methicillin‐resistant S. aureus*) strains, and inhibition zone diameters of 10 and 12 mm were reported [82]. In the present study, the sample prepared at 8 mg/mL produced an inhibition zone of 10.75 mm against *S. aureus*. Additionally, aqueous leaf extracts of *E. heterophylla* have been reported to exhibit antibacterial activity against enteric bacteria (*S. typhi, S. flexneri, E. coli*, and *P. vulgaris*). Against *E. coli*, inhibition zones of 3.33, 7.00, 9.33, and 9.67 mm were observed at concentrations of 50, 100, 150, and 200 mg/mL, respectively [83]. In the present study, the inhibition zone of 11.10 mm observed against *E. coli* at 8 mg/mL further supports the antibacterial potential of Ult‐ER under the applied experimental conditions. In another study, the methanolic extract of *E. grantii* exhibited stronger inhibition against Gram‐negative bacteria (*Shigella dysenteriae, Salmonella typhi*, and *Klebsiella pneumoniae*) and more limited activity against Gram‐positive strains (*S. aureus* and *Streptococcus epidermidis*) [84]. These findings indicate that antibacterial efficacy among *Euphorbia* species varies depending on the species, extraction solvent, and the tested bacterial strain. Similarly, the antibacterial activity of the methanolic extract of *E. cactus* Ehrenb. was investigated against Gram‐positive bacteria (*Bacillus subtilis* and *Streptococcus pneumoniae*) and Gram‐negative bacteria (*P. aeruginosa* and *E. coli*). At a concentration of 250 mg/mL, the extract produced inhibition zones of 8.4, 9.5, 11.9, and 10.1 mm against *B. subtilis, S. pneumoniae, P. aeruginosa*, and *E. coli*, respectively [85]. In the present study, the aqueous extract prepared at 8 mg/mL produced inhibition zones of 11.10 mm against *E. coli* and 10.25 mm against *P. aeruginosa*, demonstrating antibacterial activity against both tested Gram‐negative strains. Overall, when the present findings are evaluated alongside the existing literature, it can be concluded that the antibacterial potential of *Euphorbia* species varies depending on the plant species, extraction method, solvent type, and target microorganism. Nevertheless, the genus appears to represent a promising natural source of antibacterial agents against both Gram‐positive and Gram‐negative bacteria. Future studies incorporating broth microdilution‐based minimum inhibitory concentration (MIC) and minimum bactericidal concentration (MBC) determinations, broader bacterial panels, and bioactivity‐guided fractionation may further characterize the antibacterial potential observed for Ult‐ER.

### 3.5 Evaluation of the AChE and BChE Inhibitory Activity of Ult‐ER Extract

Plant‐derived sources capable of effectively neutralizing ROS have frequently been reported to exhibit enzyme inhibitory activity against various biological targets. AChE, which plays a central role in cholinergic signal transmission, is primarily localized in excitable cells of the central nervous system, whereas BChE displays a broader distribution in both central and peripheral tissues. Both enzymes are responsible for the hydrolysis of acetylcholine in the synaptic cleft, and dysregulation of this process is directly associated with cognitive decline in neurodegenerative disorders such as Alzheimer's disease. Therefore, suppression of AChE and BChE activity represents a key pharmacological strategy for preserving cholinergic transmission [50]. In recent years, due to the potential adverse effects of synthetic inhibitors, the discovery of plant‐derived, biocompatible natural enzyme inhibitors has attracted increasing attention. This pursuit is not limited to neurodegenerative diseases but is also of considerable importance for the prevention and management of prevalent chronic conditions such as diabetes, hypertension, and various dermatological disorders.

In this context, the inhibitory effects of the aqueous extract obtained from *E. macroclada* roots by UAE (Ult‐ER) on AChE and BChE enzymes were evaluated (Table 3). The results showed that Ult‐ER inhibited both enzymes, with IC_50_ values of 507.69 ± 17.95 mg/mL for AChE and 22.41 ± 0.49 mg/mL for BChE. The approximately 22.7‐fold lower IC_50_ value obtained for BChE indicates a distinct BChE‐preferential inhibitory profile under the applied assay conditions, supporting further investigation of the bioactive constituents responsible for this activity. BChE‐preferential inhibitory profiles have also been reported for plant extracts, and polyphenol‐rich extracts are known to exhibit more pronounced interactions with BChE [86, 87]. In addition, tacrine was used as a positive control in this study, and its inhibitory effects on AChE and BChE were determined as 4.33 ± 0.09 and 3.56 ± 0.05 µM, respectively. The lower inhibitory potency of the plant extracts relative to tacrine is an expected outcome. This can be explained by the fact that tacrine, as a single, chemically defined compound, exhibits high specificity and strong binding affinity toward the target enzymes, whereas plant extracts are complex mixtures containing numerous constituents, of which only a limited number are likely to exert pronounced inhibitory effects on the enzymes in question.

**TABLE 3:** Inhibitory activity of Ult‐ER (IC_50_ values) against AChE and BChE enzymes.

Sample and standard	AChE		BChE	*R* ^2^
	IC_50_ (mg/mL)	*R* ^2^	IC_50_ (mg/mL)	
Ult‐ER	507.69 ± 17.95	0.9919	22.41 ± 0.49	0.9915
Tacrine^a^	4.33 ± 0.09	0.9714	3.56 ± 0.05	0.9898

^a^ Tacrine: standard inhibitor for AChE and BChE (µM).

In the advanced stages of Alzheimer's disease, AChE activity declines whereas BChE activity increases, and BChE becomes more predominant in cholinergic regulation within the brain. For this reason, BChE‐selective inhibitors have recently been considered pharmacologically more advantageous candidates, particularly in the context of advanced Alzheimer's pathology [88]. In this regard, the strong inhibitory effect of the *E. macroclada*‐derived Ult‐ER extract on BChE suggests that the extract may serve as a supportive natural agent in neurodegenerative processes.

To date, no reports have been identified regarding the cholinesterase inhibitory activities of aqueous extracts obtained from *E. macroclada* roots using the UAE method, rendering the present findings novel in this respect. Nevertheless, studies conducted on different plant parts of various *Euphorbia* species indicate that this genus may possess cholinesterase inhibitory potential. In a study by Pisano et al.  [23], aqueous and ethanolic extracts obtained from different morphological parts of *E. characias* exhibited AChE inhibitory activity in all samples; however, only a limited number of extracts were able to inhibit BChE. Similarly, in another study evaluating the latex and aerial parts of *E. characias*, the strongest inhibitory activity was observed in the ethanolic leaf extract, with IC_50_ values of 0.6 and 390 0.39 mg/mL for AChE and BChE, respectively, whereas aqueous extracts and certain plant parts were reported to be ineffective against BChE [33]. On the other hand, in a comprehensive comparative study of methanolic extracts from different *Euphorbia* species, BChE inhibitory activity was generally found to be more pronounced than AChE inhibition; however, *E. macroclada* did not exhibit significant inhibition against AChE [53]. In the present study, although the IC_50_ value of the Ult‐ER extract against AChE was determined to be 507.69 ± 17.95 mg/mL, the IC_50_ value calculated for BChE was markedly lower at 22.41 ± 0.49 mg/mL, clearly demonstrating a pronounced and selective inhibitory potential of *E. macroclada* roots toward BChE. Considering that BChE inhibition reported for many *Euphorbia* species is limited or strongly dependent on the extraction solvent, the potent activity observed here may be attributed to root‐specific bioactive constituents, as well as to the ability of UAE to enhance cell wall disruption and improve the recovery of polar compounds. In this respect, Ult‐ER exhibits a remarkable bioactive profile among *Euphorbia* species, surpassing previously reported findings, particularly in terms of BChE‐targeted cholinesterase inhibition.

Ult‐ER exhibited inhibitory activity against both cholinesterase enzymes, with a clearly lower IC_50_ value for BChE than for AChE, revealing a BChE‐preferential inhibitory profile under the applied assay conditions. Considering the increasing relevance of BChE to cholinergic regulation during the progression of Alzheimer's disease, this finding highlights *E. macroclada* roots as a promising source of bioactive constituents for further investigation. To the best of our knowledge, the present study provides the first evaluation of the cholinesterase‐inhibitory activity of an ultrasound‐assisted aqueous extract obtained from *E. macroclada* roots, thereby offering a novel basis for bioactivity‐guided fractionation and compound isolation studies.

The present study was designed as an initial in vitro characterization of the biological potential of Ult‐ER. The spectrophotometric TPC, TFC, and antioxidant assays provided complementary information on the overall response of the extract, while future chromatographic and spectrometric profiling may enable the identification of the individual constituents associated with these activities. Similarly, the antibacterial potential demonstrated by the agar well diffusion assay may be further characterized through broth microdilution‐based MIC and MBC determinations. The observed BChE‐preferential inhibitory profile also provides a basis for subsequent enzyme‐kinetic, cell‐based, and in vivo investigations. These complementary approaches may further clarify the active constituents and expand the biological and potential pharmaceutical applications of Ult‐ER.

## 4 Conclusion

In conclusion, this study provides, to the best of our knowledge, the first integrated in vitro evaluation of the antioxidant, antibacterial, and cholinesterase‐inhibitory properties of an ultrasound‐assisted aqueous extract obtained from *E. macroclada* roots. Ult‐ER displayed a BChE‐preferential inhibitory profile, with a lower IC_50_ value for BChE (22.41 ± 0.49 mg/mL) than for AChE (507.69 ± 17.95 mg/mL). The extract also exhibited measurable responses in complementary antioxidant assays and antibacterial activity against all four tested bacterial strains at 8 mg/mL, producing inhibition zones ranging from 10.25 to 11.10 mm. Together with the TPC and TFC findings, these results highlight *E. macroclada* roots as a promising source of biologically active constituents and demonstrate the value of ultrasound‐assisted aqueous extraction for their recovery. Future studies involving chemical profiling, bioactivity‐guided fractionation, enzyme‐kinetic and cell‐based analyses, MIC and MBC determinations, and in vivo validation may further clarify the constituents responsible for these activities and expand the potential applications of Ult‐ER.

## Author Contributions

**Abdulkadir Gül**: conceptualization, methodology, investigation, data curation, formal analysis, and writing – original draft. **Fırat Yılmaz**: methodology, validation, and formal analysis. **Fevzi Topal**: methodology, data interpretation, and writing – review and editing. **İnanç Özgen**: taxonomic identification, investigation, and resources. **Ercan Aydoğmuş**: methodology, validation, supervision, and writing – review and editing. **Hasan Arslanoğlu**: conceptualization, supervision, and writing – review and editing. All authors have read and approved the final version of the manuscript.

## Funding

The authors have nothing to report.

## Consent to Participate

The authors agreed to participate in this work.

## Consent to Publish

The authors agreed to publish this work.

## Ethics Statement

The authors have nothing to report.

## Conflicts of Interest

The authors declare no conflicts of interest.

## Data Availability

The data that support the findings of this study are available from the corresponding author on request.
